# Opportunistic citizen science data reveals habitat selection of lesser black‐backed gulls in Central Europe

**DOI:** 10.1002/ece3.10802

**Published:** 2023-12-06

**Authors:** Zsuzsanna Lánczos, Zsolt Végvári, Zoltán Ecsedi, Emil Boros

**Affiliations:** ^1^ Doctoral School of Biology, Institute of Biology Eötvös Loránd University Budapest Hungary; ^2^ Institute of Aquatic Ecology Centre for Ecological Research Budapest Hungary; ^3^ Senckenberg Deutsches Entomologisches Institut Müncheberg Germany; ^4^ Hortobágy Environmental Association Balmazújváros Hungary

**Keywords:** bird ecology, birdwatching, Hungary, land cover, macroecology

## Abstract

This study is the first attempt to find a relation between the opportunistic observation data of an easily identifiable but locally rare bird, in our case the lesser black‐backed gull (*Larus fuscus fuscus*), and the Corine Land Cover habitat map by applying a resource selection function model to reveal the habitat selection of the species. We provide a scientific analysis using the valuable citizen science dataset sourced from the www.birding.hu website, collected by hundreds of volunteers over a span of more than a decade in Hungary. Birds selected for landfills, river and lake habitats and showed a significant but much smaller attraction towards urban areas, while they selected against arable lands. We found that in the case of rare and moderately common birds, we can obtain a representative picture of the habitat selection of the species, even if the data are collected by non‐standardized means.

## Graphical abstract

In this study, we show that opportunistic observation data of an easily identifiable but locally rare bird, in our case the lesser black‐backed gull (*Larus fuscus fuscus*), can be used to reveal the habitat selection of the species. Birds selected for landfills, river, and lake habitats and showed a significant, but much smaller attraction towards urban areas, while they selected against arable lands.

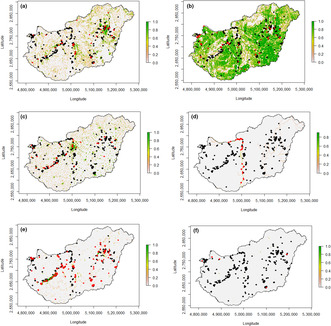

## 1 INTRODUCTION

The number of scientific publications using data collected by citizen scientists has been continuously increasing over the past 15 years (Bergerot, 2022). Compared to other disciplines, volunteers are of key importance for ornithology (Nicholson, 1959). Research using data collected by birders devoting their leisure time to their passion and being also present in remote areas has played a significant role in ornithology, and it is expected to continue to do so in the future (Greenwood, 2007).

Like most European countries, Birdlife Hungary has a large body of hobbyist experts who have long studied birds and who are active on a broad geographic scale at a national level. In general, birds are easy to be observed and counted compared to other groups of animals (Gill, 2006). Besides, citizen science is considered to be particularly effective at finding rare species (Dickinson et al., 2010). Such is the case with the lesser black‐backed gull (*Larus fuscus fuscus*), hereinafter referred to as LBG, which appears only sporadically in Hungary (http://www.mme.hu/magyarorszagmadarai/madaradatbazis‐larfus, 2022) and what we chose as an easily identifiable target species in our study.

No other field of ecology depends more on data collected by citizen scientists than macroecology, which studies the relationships between organisms and their environments at large spatial scales to characterize and explain statistical patterns of abundance, distribution and diversity (Brown, 1995; Gaston & Blackburn, 2000). Here, we aim to investigate the habitat selection of LBGs in Hungary based on birders' observations.

Breeding populations of gulls in urban environments have undergone rapid population growth worldwide (Spelt et al., 2019). Today, urban nesting by large gulls is widespread and has been observed in various European countries, including Bulgaria, France, Denmark, Italy, Belgium, Norway, Portugal, Finland, Estonia, Latvia, Croatia, Ireland and Britain (Rock, 2005). A majority of studies investigating the effects of urbanization indicate an increase in bird density, coupled with a decrease in species richness and evenness. This shift is primarily attributed to augmented food availability (Marzluff, 2001). Opportunistic and omnivorous, LBGs take advantage of a wide range of nutrition resources (Rock, 2005). From the 1970s to the 2000s, the urban nesting population of LBGs in the United Kingdom, for example, witnessed significant growth, allowed by increased food abundance, high reproductive success and seemingly unbounded habitats for breeding (Rock, 2005).

Movement patterns and habitat use of gulls have been studied by GPS‐based tracking devices in near‐sea regions of Europe (Camphuysen et al., 2015; Langley et al., 2022; Spelt et al., 2019). A study in the United Kingdom, which combined Global Positioning System (GPS) tracking data of 12 urban‐nesting LBGs (*Larus fuscus*) with habitat data, found that despite the proximity of the sea, urban nesting gulls made significant use of the terrestrial environment only, spending two‐thirds of their time in suburban and urban areas and one‐third in rural green areas (Spelt et al., 2019). Gulls tended to increasingly use anthropogenic food resources, including fisheries discards, landfills and agricultural lands (Duhem et al., 2008; Navarro et al., 2010), based on studies in Spain and France. Garthe et al. (2016) found that LBGs breeding at the coast of the southern North Sea used the anthropogenic landscape intensively. On land, gulls feed primarily on bare soil, with significantly higher potato land use and significantly less grassland use. In the Netherlands, predominantly terrestrial habitats were used by LBGs, breeding 30 km from the coast. Besides landfills, as the most popular habitat, agricultural fields and freshwater bodies were also used for foraging (Gyimesi et al., 2016). Landfills have long been known to be food sources for breeding and migrant gulls (Belant et al., 1998; Cramp & Simmons, 1983). However, it is still a question to be answered as to how important they actually are (Belant et al., 1998; Rock, 2005).

Despite being a seabird, the LBG appears sporadically in Hungary as well, especially in autumn and spring (http://www.mme.hu/magyarorszagmadarai/madaradatbazis‐larfus, 2022). During their migration, they stop frequently and for a relatively longer time to forage (Klaassen et al., 2012). Previous studies on habitat use and selection with GPS‐based tracking devices covered near‐sea areas (Camphuysen et al., 2015; Garthe et al., 2016; Gyimesi et al., 2016; Isaksson et al., 2015; Spelt et al., 2019). The main goal of this study was to analyse the opportunistic citizen science data of LBGs in order to reveal their habitat selection in Hungary, which is a landlocked country, hundreds of kilometres from the nearest coast.

## 2 MATERIALS AND METHODS

### 2.1 Study region

Hungary, our study area, is a landlocked country in Central Europe, spanning 93,030 square kilometres of the Carpathian Basin, which almost entirely belongs to the Danube catchment area. Most of the country has an elevation under 200 m (The World Factbook, 2022). It is traditionally divided into six major geographic regions, which can be further divided into 35 medium‐sized regions (Fésűs & Nagy, 2005). The country's climate can be best characterized as humid continental.

### 2.2 Data source

Observation data of adult and subadult LBGs collected by Hungarian ornithologists and bird watcher enthusiasts between 2004 and 2021 and recorded on the birding.hu website (https://www.birding.hu, 2022) formed the basis of our analysis for the habitat selection of LBGs in Hungary. The website, which has 8893 unique registered users, is the largest citizen science project in Hungary and serves as a central repository where 315,744 observations have been submitted (till 15‐Dec‐2022) since its foundation in May 2004. Birders who are driven by a healthy competitive spirit are traditionally encouraged by the website to report rarities. The database retrieved from the www.birding.hu website was supplemented with a series of data collected by the Hortobágy Environmental Association (HEA) and Hortobágy National Park Directorate (HNPD) from the wider area of the Hortobágy National Park and provided to us. Members of the HEA and employees of the HNPD collect data on bird rarities in the area primarily for their own scientific use.

Professional birdwatchers (biologists, national park employees, rangers and Birdlife Hungary staff) as well as hobby birders with a high level of species recognition skills provided the data. Due to the unambiguous identification of adult and subadult LBGs, our approach relies on the assumption that there are no identification errors, or that they are very rare; thus, we consider the result to be unaffected by misidentification. We can confidently make this assumption because, on the territory of Hungary, no other members of the Laridae family can be confused with the locally occurring *Larus fuscus fuscus* subspecies in its adult and subadult states due to its unique black back. The only local species similar to the adult and subadult LBG is the great black‐backed gull (*Larus marinus*), but it is larger, has a more robust beak and has flesh‐coloured legs. Based on the observers' comments, missing coordinates were added, some locations were clarified, and duplications were deleted. This led to 1276 observations of 3186 individual adult or subadult birds, recorded by 102 different birdwatchers.

The data were collected by non‐standardized means, with no sampling design and no standardized protocol. The amount of this so‐called opportunistic data are increasing more and more with the growing popularity of citizen science (Dickinson et al., 2010, 2012; Hochachka et al., 2012). According to Tulloch and Szabo (2012), observer activity is typically concentrated in certain habitat types, resulting in uneven distribution of observation efforts across different environments. However, the observers in our case are birdwatchers who conduct their observations during their daily routine activities and in their free time as well, and who are scattered throughout the country. As Cretois et al. (2021) showed, it is possible to infer habitat selection for a species in an area rich in opportunistic data. Besides, if opportunistic data are abundant, the gain in accuracy can be considerable, especially in the case of rare species (Giraud et al., 2016). Being only sporadically present in the country, the LBG should be considered as a rare, though easily detectable, non‐cryptic species in Hungary.

### 2.3 Habitats

**FIGURE A1:**
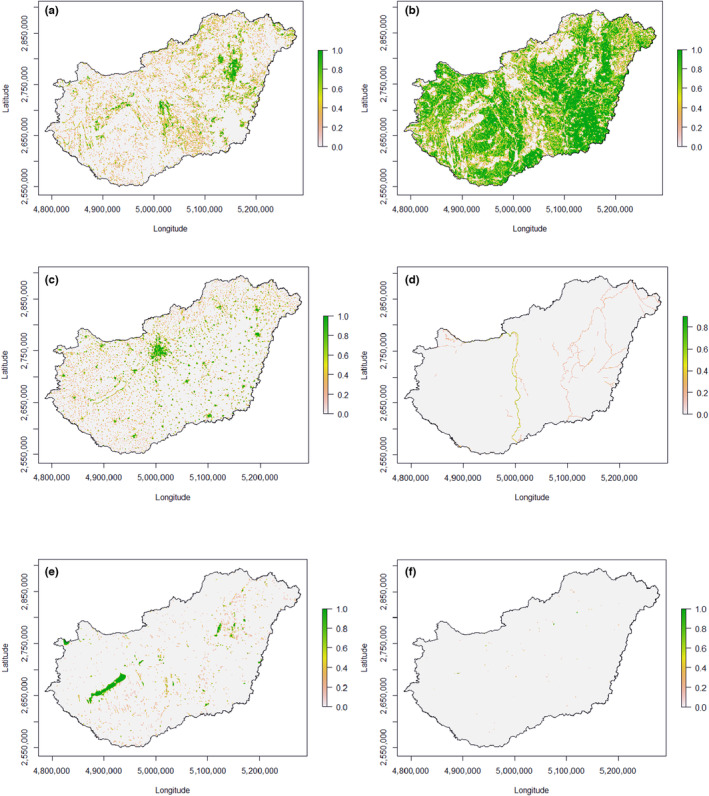
Spatial distribution of habitat types ((a) grasslands, (b) arable lands, (c) urban areas, (d) rivers, (e) lakes and (f) landfills) in Hungary. 1 means that the area within a circle with a radius of 500 m drawn around the centre of a grid cell is 100% covered by (a) grasslands or (b) arable lands or (c) urban areas or (d) rivers or (e) lakes or (f) landfills. The map was made in the standard European Coordinate Reference System – ETRS89/LAEA1052.

All statistical analyses were conducted in R version 4.3.0 (R Core Team, 2023). The habitat map was based on the 100 ‐m resolution Corine Land Cover European database (European‐Commission. Corine Land Cover, 2012. http://land.copernicus.eu/pan‐european/corine‐land‐cover/clc‐2012 2016). We regrouped the 44 habitat types of Corine into 7 main habitat types. As a result, the following habitat types were obtained: (1) arable land; (2) urban area; (3) grassland; (4) landfills; (5) lakes; (6) rivers and (7) other – the last being unsuitable for foraging (e.g., forest). Subsequently, a 500‐metre buffer was created around the centre of each raster pixel, and we calculated the percentage of each habitat type represented in this circle of 500‐m radius. We thus obtained seven values for each pixel according to the habitat types. These values were then used to generate a raster stack comprising seven layers, each representing one of the defined habitat types. The pixel values of each raster layer indicate the proportion of each habitat type for the 500‐m radius circle surrounding the centre of the pixel (Appendix A: Figure A1). By employing this approach, we transitioned from categorical to numerical data, thereby gaining information about the overall composition of the space surrounding the observed animal (Street et al., 2016). The habitat information was extracted from the proportional maps and assigned to the GPS coordinate of each observation using R version 4.3.0 (R Core Team, 2023).

**FIGURE 1:**
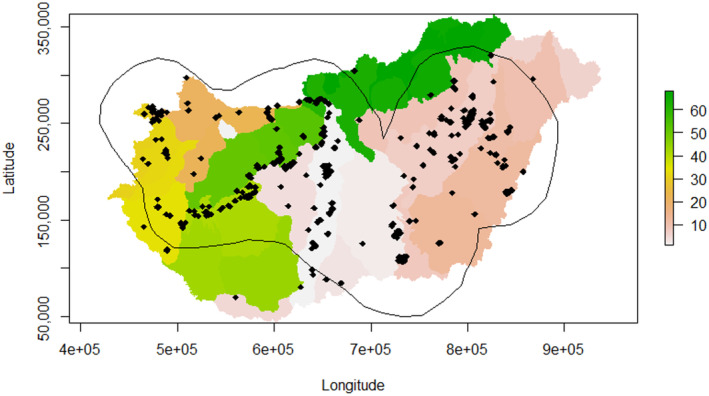
Map of medium‐sized regions of Hungary with adult and subadult lesser black‐backed gull observation points and 95% KDE home range in HU_HD72/EOV coordinate system.

In habitat selection studies, like resource selection functions (Boyce & McDonald, 1999), that we used in our analysis, in addition to ‘used’ (presence) locations, ‘available’ (pseudo‐absence) locations should also be taken into consideration (Manly et al., 2007). We are aware that most of the studies using resource selection functions are applied to animal location data obtained from telemetry technologies. However, it is important to note that use‐availability approaches can be extended to other types of data, such as snow tracking, aerial survey or observation data, etc. (Manly et al., 2007). In the case of mammals, theoretically available places are not always accessible. This, however, is not the case for birds, which possess the capacity to fly over geographical barriers that might impede mammals. Hence, precision in defining what we consider available is crucial in habitat selection studies and may affect the quantification of selection (Beyer et al., 2010). To avoid under‐sampling, we systematically sampled (Fieberg et al., 2021) available points from within a 95% probability contour from kernel density estimators (KDE) applied to the observation data (Fieberg & Börger, 2012), thereby extracting the habitat information from each pixel of the home range of the population. To control for potential spatial autocorrelation, another variable, the medium‐sized geographical region, was included in the model construction (Marosi & Somogyi, 1990) (Figure 1).

### 2.4 Statistical analysis

The evaluation process was implemented by fitting a mixed‐effect RSF (logistic regression) model (Muff et al., 2020) using the ‘*lme4’* package (Bates et al., 2015) in R. The medium‐sized geographical region was treated as a random effect in the model, while the proportional habitat type values were treated as fixed factors (Gillies et al., 2006). We assessed the degree of collinearity by calculating variance inflation factors (VIF) (Graham, 2003). One variable, the grassland proportional coverage, had a collinearity problem, so it was excluded from the model construction. Adhering to the recommendations of Fithian and Hastie (2013), we assigned weights of 1 to used and 5000 to available points. To avoid spatial distortion, all statistical analyses were performed while maintaining the raster maps in their original coordinate system.

Additionally, we sought to explore any potential correlation between LBG sightings and human population density. This investigation was driven by our interest in understanding how the presence of a larger human population, potentially leading to enhanced bird detection, might impact the outcomes of our habitat selection analysis utilizing opportunistic citizen science observation data. To achieve this, NASA's Gridded Population of the World map of 2020 at 30 arc‐second resolution (CIESIN, 2018) was used, and a generalized linear mixed effect model was performed for the statistical analysis. We conducted a reiteration of the previous analysis, this time also incorporating human population density as a variable.

## 3 RESULTS

**FIGURE 2:**
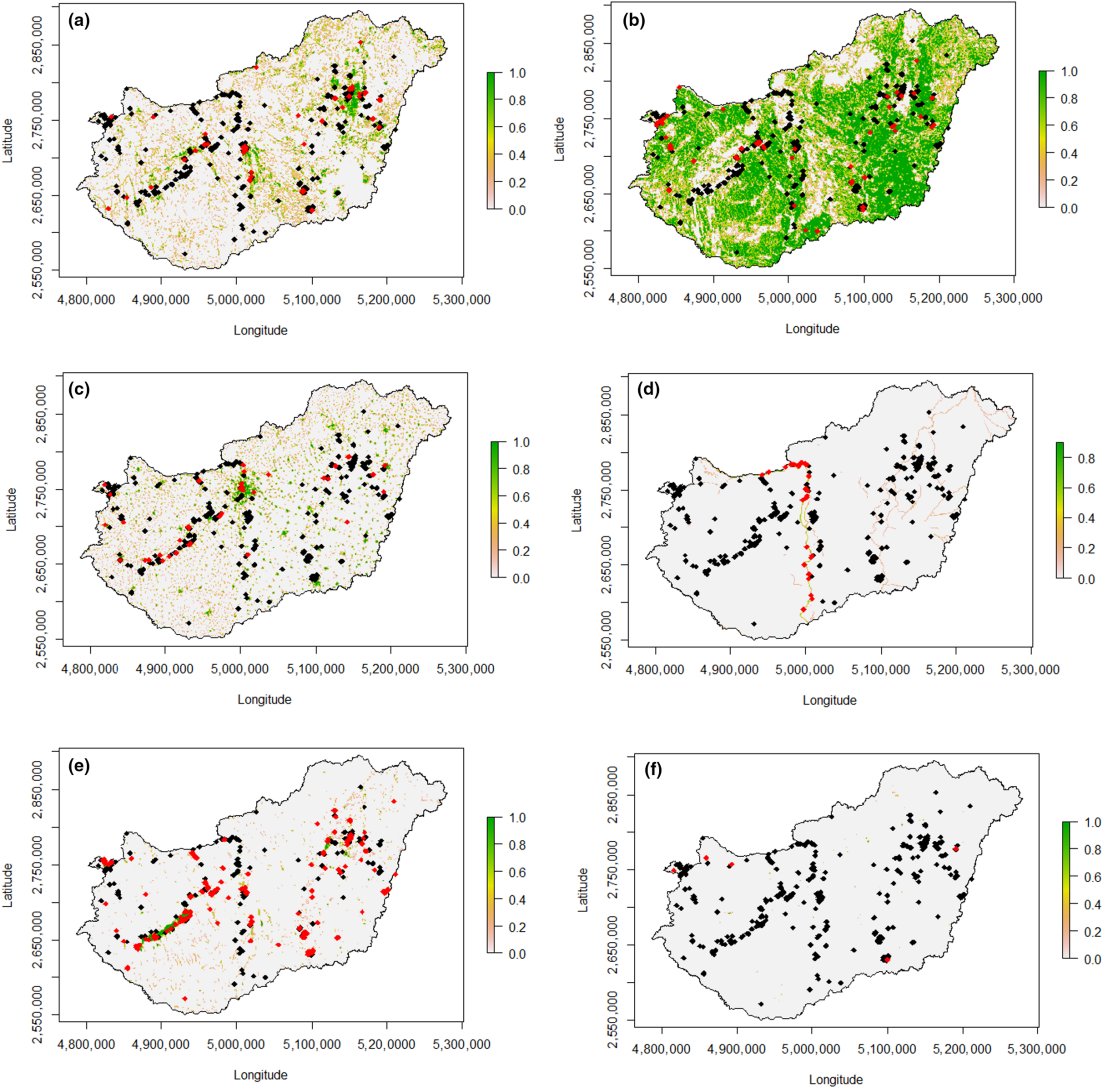
Spatial distribution of observation records of adult and subadult lesser black‐backed gulls (*Larus fuscus fuscus*), projected onto (a) grasslands, (b) arable lands, (c) urban areas, (d) rivers, (e) lakes and (f) landfills in Hungary. 1 indicates that the area within a circle with a radius of 500 m drawn around the centre of a grid cell is 100% covered by (a) grasslands or (b) arable lands or (c) urban areas or (d) rivers or (e) lakes or (f) landfills. The red crosses indicate observations made in (a) grasslands or (b) arable lands or (c) urban areas or (d) rivers or (e) lakes or (f) landfills, respectively (according to Corine habitat types) and the black crosses indicate observations in all the other habitats. The map was made in the standard European Coordinate Reference System – ETRS89/LAEA1052.

**FIGURE 3:**
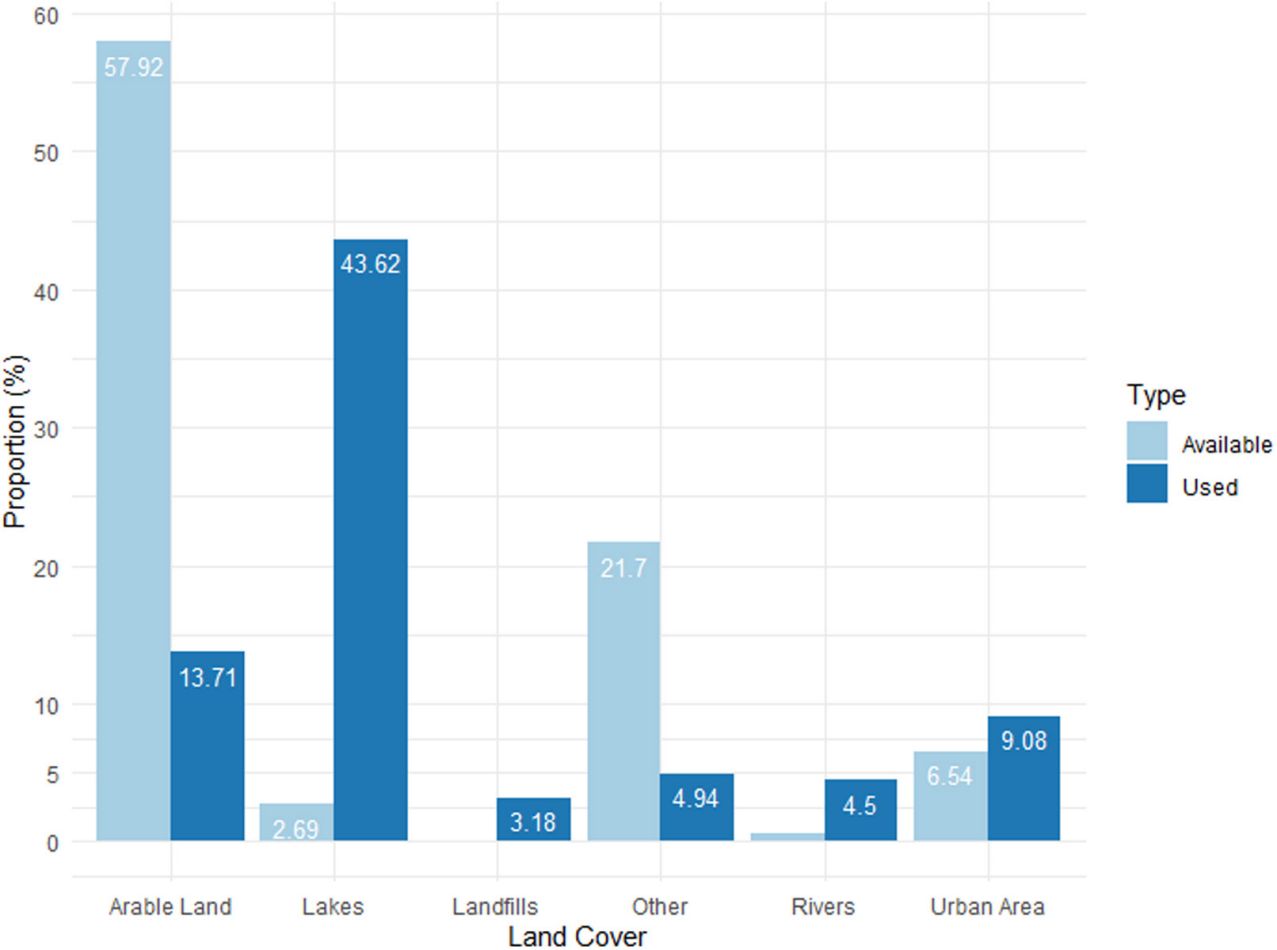
Distribution of used and available locations among different landscape cover classes for adult and subadult black‐backed gulls. The bar chart of the different habitat types show the average percentage of the 500 m radius circles drawn around the centre of each raster cell by the given habitat types.

Despite having only a few observations (*n* = 97 with 205 birds) in landfills, the used model indicated a strong selection for this habitat type (*β*_landfills_ = 5.84). The estimated coefficient for rivers, with only a few sightings (*n* = 88 with 118 birds), was also in the positive direction (*β*_rivers_ = 4.524). LBGs also selected (*β*_lakes_ = 4.46) for lakes, where most of the observation data (*n* = 661) were collected, with a total of 1460 birds. Although more sightings were recorded in urban areas (*n* = 133 with 411 birds) than in landfills and rivers, our statistical model, considering not only the used but also the available areas, showed a weaker, slightly positive selection for this habitat type (*β*_urban area_ = 0.631). In the case of arable lands, the total number of birds observed (601 on 105 occasions) was high, but according to our analysis, LBGs selected against them (*β*_arable land_ = −2.204) (Figures 2 and 3; Tables 1 and 2).

**TABLE 1:** Number of observations and number of observed lesser black‐backed gulls (*Larus fuscus fuscus*) in different habitats.

	Number of observations	Number of individuals
Lakes	661	1460
Urban area	133	411
Grassland	173	314
Landfills	97	205
Arable land	105	601
Rivers	88	118
Other	19	77
Total	1276	3186

**TABLE 2:** Results of the used generalized linear mixed effect model.

	Fixed effects	Estimate SE	z value	Pr(>|*z*|)
Intercept	−18.243	0.062	−295.76	<.001
Landfills	5.84	0.063	92.68	<.001
Rivers	4.524	0.056	80.35	<.001
Lakes	4.46	0.064	70.21	<.001
Urban area	0.631	0.06	10.55	<.001
Arable land	−2.204	0.0467	−47.26	<.001

Upon scrutinizing the relation between LBG sightings and human population density, including it as an additional variable in the analysis, we did identify a significant correlation between the two values. However, this correlation did not markedly alter the habitat selection preferences (Table 3). Consequently, we have justifiable confidence that the data are not subject to bias. This correlation likely emanates from the urbanization patterns observed in gull populations.

**TABLE 3:** Results of the used generalized linear mixed effect model with human population density.

	Fixed effects	Estimate SE	*z* value	Pr(>|z|)
(Intercept)	−18.24	0.125	−145.49	<.001
Human population density	0.0001	0.00001	8.853	<.001
Landfills	5.806	0.155	37.573	<.001
Lakes	4.444	0.076	58.485	<.001
Rivers	4.427	0.14	31.644	<.001
Urban area	0.387	0.1	3.849	<.001
Arable land	−2.191	0.093	−23.634	<.001

## 4 DISCUSSION

The vast amount of opportunistic citizen science data stored in various repositories (Tewksbury et al., 2014) has promising transformative potential in the field of ecology (Bela et al., 2016). According to our knowledge, our work is the first attempt to provide a statistical analysis of the valuable dataset uploaded to the regional birdwatching (www.birding.hu) website (as well as the HEA and HNPD database), collected by hundreds of volunteers for more than a decade. This citizen science data allowed us to reveal the habitat selection of LBGs in a land far from the sea.

In support of our ecological predictions, LBGs, just like most birds, moved towards habitats with abundant food. Birds selected for landfills, rivers, and lakes and showed a significant, but much smaller preference towards urban areas and selected against arable lands.

As noted by Duhem et al. (2008) and Navarro et al. (2010), gulls prefer anthropogenic food sources, such as landfills, based on studies conducted in Spain and France. Similarly, in a study by Gyimesi et al. (2016), besides landfills as the most popular habitat, agricultural fields and freshwater bodies were also used for foraging by LBGs in the Netherlands. In our own investigation, landfills proved to be the most preferred habitat types, followed by rivers and lakes. However, in contrast to the above‐mentioned previous findings, LBGs observed in Hungary showed a selection against arable lands. This distinction may be attributed to the continued prevalence of landfilling practices in the eastern parts of Europe (https://www.europarl.europa.eu/, 2022), resulting in superior nutrient supply compared to arable lands. The heavy selection of LBGs for rivers in our study can be explained by their degraded state. Specifically, only 11% of watercourses in Hungary demonstrate excellent or good ecological status, while the majority (89%) fall below these standards. Furthermore, a mere 50.9% of water bodies in watercourses achieved a good chemical state (Ministry of the Interior of Hungary, 2021). Based on our observations, the disposal of organic material from cargo ships into the Danube remains a recurrent occurrence, rendering river habitats highly attractive to gulls, including the LBG.

We suggest that the data from www.birding.hu holds potential for analysing habitat selection of other species within the Hungarian avifauna. Given the constraints of this kind of data collection method, it is most suitable for studying rare and moderately common species that are easily identifiable. The LBG featured in this study is precisely such a distinctive, easily recognizable species with its unmistakeable black back, setting it apart from other Hungarian gulls. On the other hand, it is rare enough to arouse the interest of birders and encourage them to record the observation in the online database, which can therefore considered representative. We acknowledge the inherent limitations of such opportunistic observation data and advocate that it should be treated with caution. Nevertheless, with due care, it yields valuable information about the studied species. Moreover, meticulous data recording, including the precise entry of GPS coordinates, can enhance the availability and quality of the dataset.

Online repositories provide an opportunity to link citizen science data collected on different species to other databases that include information on land cover, topography, census data, LIDAR (Light Detection and Ranging) and NDVI (Normalized Difference Vegetation Index) (Dickinson et al., 2010), as well as protected areas. In this study, we did not delve into the relationship between LBG observation data and protected areas, since this species is not specifically associated with conservation‐designated lands; rather, it increasingly relies on anthropogenic food resources. In addition, our research underscores that, due to the otherwise welcomed development of waste management technology, the nutrition availability for Hungarian LBGs is narrowing, which, coupled with the lack of alternative food resources, may adversely affect the species' occurrence in Hungary in the future.

Finally, it can be concluded that, opportunistic citizen science data of bird observations, if handled with due diligence, can provide a valuable data source to investigate the habitat selection of specific species.

## AUTHOR CONTRIBUTIONS

**Zsuzsanna Lánczos:** Conceptualization (equal); data curation (equal); formal analysis (equal); investigation (equal); methodology (equal); software (equal); validation (equal); visualization (equal); writing – original draft (equal). **Zsolt Végvári:** Data curation (equal); formal analysis (equal); methodology (equal); software (equal); supervision (equal); validation (equal); writing – review and editing (equal). **Zoltán Ecsedi:** Data curation (equal); resources (equal); writing – review and editing (supporting). **Emil Boros:** Conceptualization (equal); data curation (equal); formal analysis (equal); methodology (equal); resources (equal); supervision (equal); validation (equal); writing – review and editing (equal).

## CONFLICT OF INTEREST STATEMENT

The authors have no competing interests to declare.

## Data Availability

The data will be archived and made available on Dryad upon acceptance of the manuscript at the following location: https://doi.org/10.5061/dryad.z34tmpgjh. The data can now be accessed through the Dryad link: https://datadryad.org/stash/share/Pm6NkBkX8QeB_oFOY3f0Mr0Hg_Cj8‐lHzhW3J0KJ‐jA.
